# Carbon Monoxide Emission and Air Quality Analysis Based on an Improved Double-Weight Fuzzy Comprehensive Evaluation

**DOI:** 10.3389/fpubh.2021.790383

**Published:** 2022-01-14

**Authors:** Weicai Peng, Xiangguo Liu, Farhad Taghizadeh-Hesary

**Affiliations:** ^1^Department of Mathematics and Statistics, Chaohu University, Hefei, China; ^2^Department of Mathematics and Statistics, Chaohu University, Hefei, China; ^3^Social Science Research Institute, Tokai University, Hiratsuka, Japan

**Keywords:** carbon monoxide emission, air quality, double-weight fuzzy comprehensive evaluation, Hefei, pollution

## Abstract

In this article, we adopt an improved double-weighted fuzzy comprehensive evaluation method to investigate the air condition of Hefei City from July 2016 to July 2021. We focus on the impact of the toxicity index, especially the impact of carbon monoxide, which is also considered in some other kinds of quality evaluation, such as water classification. Firstly, we found that with the increasing awareness of environmental protection and with the attention of the government to the quality of air in recent years, the air conditions have become better (the grades become lower). Secondly, the value of the factors, PM_2.5_, PM_10_, SO_2_, CO, NO_2_, and O_3_ periodically fluctuate from year to year; and the periodicity of O_3_ is reversed with the other factors. Finally, the monthly average analysis shows that the overall air quality is good; all the grades are I-II, except for December 2017 which has a grade III. Furthermore, the air quality in the winter (especially in December and January) is not always good.

## Introduction

Since humanity entered the era of industrial civilization while creating an enormous material of wealth, it has also accelerated the grab of natural resources, breaking the balance of the ecosystem of the earth. The deep-seated contradiction between man and nature has become increasingly apparent. In recent years, climate change, loss of biodiversity, worsening desertification, and frequent extreme climate events have posed severe human survival and development challenges. Climate and environmental quality assessment have gradually attracted the attention of various governments.

Air quality evaluation is an essential part of the environmental quality evaluation, which is of great significance to objectively understand the status quo of urban air pollution, of the development trend of pollution, and the study of corresponding pollution control countermeasures. The air quality assessment in China mainly includes PM_2.5_, PM_10_, SO_2_, CO, NO_2_, and O_3_. Eighty-six percent of carbon monoxide in the urban atmosphere is emitted by automobiles. The emission of automobiles exhaust gas is related to the speed of the car. The higher the speed of the car, the lower the carbon monoxide emission.

Carbon monoxide also comes from the industrial production of coal. During combustion, the worse the oxygen supply condition, the higher the carbon monoxide content. Carbon monoxide emissions also occur in gas, water gas processing, and cooking. From the air, it could enter the body through the respiratory system and then, into the human blood, causing hypoxia and necrosis in body tissue, which could endanger life. Therefore, carbon monoxide is a highly toxic pollutant to the blood and nervous system (toxicity index is of the highest value 5). The following Table 1 shows the toxicity index of carbon monoxide.

**Table 1:** Toxicity index *f*_*i*_ of each factor.

**Factor**	**PM_**2.5**_**	**PM_**10**_**	**SO_**2**_**	**CO**	**NO_**2**_**	**O_**3**_**
Toxicity ***f***_***i***_	1	2	5	5	4	3

Some known mathematical methods, including the artificial neural network method (1), gray clustering method (2), and fuzzy comprehensive evaluation method (3, 4), are widely applied in some areas, such as water quality evaluation (5, 6), coal quality evaluation (7), and air quality evaluation (8–11). Generally, air quality evaluation methods include fuzzy clustering classification, gray cluster correlation analysis, green air pollution comprehensive index, and fuzzy comprehensive evaluation methods. In the classical work, Chelani and Devotta (12) adopted a non-linear dynamical theory to analyze the PM_10_ in Mumbai of India. The results show that the prediction relative error of the non-linear local approximation model is greater than that of the autoregressive model, thus, the prediction relative error of the non-linear local approximation model is better than that of the autoregressive model (13). The definition of air quality level is fuzzy, and the fuzzy comprehensive evaluation method is a comprehensive evaluation method based on fuzzy mathematics. The fuzzy comprehensive evaluation of air quality fully considers the interaction of various pollutant factors. The evaluation process is not affected by the time, space, and type of pollutant factors, and can effectively control the evaluation error and make the evaluation result more in line with the actual situation.

The fuzzy comprehensive evaluation method is widely used in air quality evaluation. However, most studies simply use it to evaluate the air quality of a particular region, while a few studies combine the fuzzy comprehensive evaluation method with other methods to make the fuzzy comprehensive evaluation method more reasonable. Li and Li (14), based on over-standard weighting, claimed that the toxicity of evaluation factors was considered to construct a double-weight fuzzy comprehensive evaluation model and to evaluate the air quality of Nanjing. The results show that their evaluation method with toxicity is more objective than the traditional fuzzy comprehensive evaluation method (14). Based on the fuzzy comprehensive evaluation method, Luo (15) has selected the annual air quality data of 10 Chinese cities as evaluation factors to evaluate the air quality of these ten cities (16). In a study by Zhao et al. (17), the fuzzy comprehensive model based on entropy technology for air quality assessment was established by improving the method of computing the weights of factors (17). He (9) analyzed the air quality of Jingzhou City according to the air quality index and the concentration of various pollutants from 2014 to 2018. He (9) also studied the relationship between the air quality of Jingzhou City, meteorological factors, and the surrounding cities by using correlation analysis and regression analysis methods. Double weight fuzzy comprehensive evaluation method, fuzzy comprehensive evaluation method with double slope function, and double weight fuzzy comprehensive evaluation method with double slope function are applied in air quality evaluation of Jingzhou City (9). By applying the fuzzy comprehensive evaluation method, Liang et al. (18) analyzed the concentration distributions of the particles in the air and were discussed based on 31 different particle diameters (18). Zhang et al. (19) analyzed the possible regional source of the PM_2.5_ mass concentrations in Beijing of China by applying the Potential Source Contribution Function (PSCF) method. Their investigation shows that regional sources in different seasons could be one of the crucial contributors to PM_2.5_ mass concentrations (20).

However, when fuzzy comprehensive evaluation is adopted to an environmental quality evaluation, the construction of the membership function also has arbitrariness. That is, it does not satisfy additivity. Especially when there are many pollutant factors and the weight definition and selection of evaluation factors are different, it is easy for the phenomenon to appear to be of unclear grading and unreasonable results, which leads to the deviation of evaluation results. Therefore, this article adopts an improved double-weighted fuzzy comprehensive evaluation (ID-FCE) method to emphasize the impact of toxicity, to evaluate the air situation in Hefei city of Anhui Province based on the air quality index, and to identify the concentration of various pollutants in Hefei from July 2016 to July 2021.

Our contributions to the literature are 2-fold. First, from the aspect of research methodology, we adopt an improved double-weighted fuzzy comprehensive evaluation method through generalizing the known fuzzy comprehensive evaluation method to investigate the impact of the toxicity index—especially the impact of carbon monoxide, which is an essential index in the automobile exhaust emission detection. In our article, the toxicity effects are aggravated as the toxicity index value increases, which is reversed in most of the existing literature. Second, from the aspect of research objectives, we focus the investigation on the data of the daily air condition of Hefei City from July 2016 to July 2021, which has not been analyzed as far as we know [Huang et al. (21) studied Hefei air condition from 2001 to 2012, see (21)].

We organize the rest of the article as follows. In section Data Descriptive Statistics, we make the descriptive statistics of our collected data. In section Improved Double-Weight Fuzzy Comprehensive Evaluation Method, we give the improved double-weighted fuzzy comprehensive evaluation method. In section Case Study, the case study is presented. In section Conclusion, we make the conclusion.

## Data Descriptive Statistics

This article uses the daily air quality data of Hefei City of Anhui Province from July 2016 to July 2021 for fuzzy evaluation analysis. The air quality index data came from the China Air Quality Online Monitoring and Analysis Platform (https://www.aqistudy.cn). We collected 1,857 days of data; the validation of which is shown in Table 2.

**Table 2:** Valid number of each factor (Total 1,857 days).

**Factor**	**PM_**2.5**_**	**PM_**10**_**	**SO_**2**_**	**CO**	**NO_**2**_**	**O_**3**_**
• Valid number • Invalid number	• 1855 • 2	• 1855 • 2	• 1857 • 0	• 1857 • 0	• 1857 • 0	• 1845 • 12

**Figure 1:**
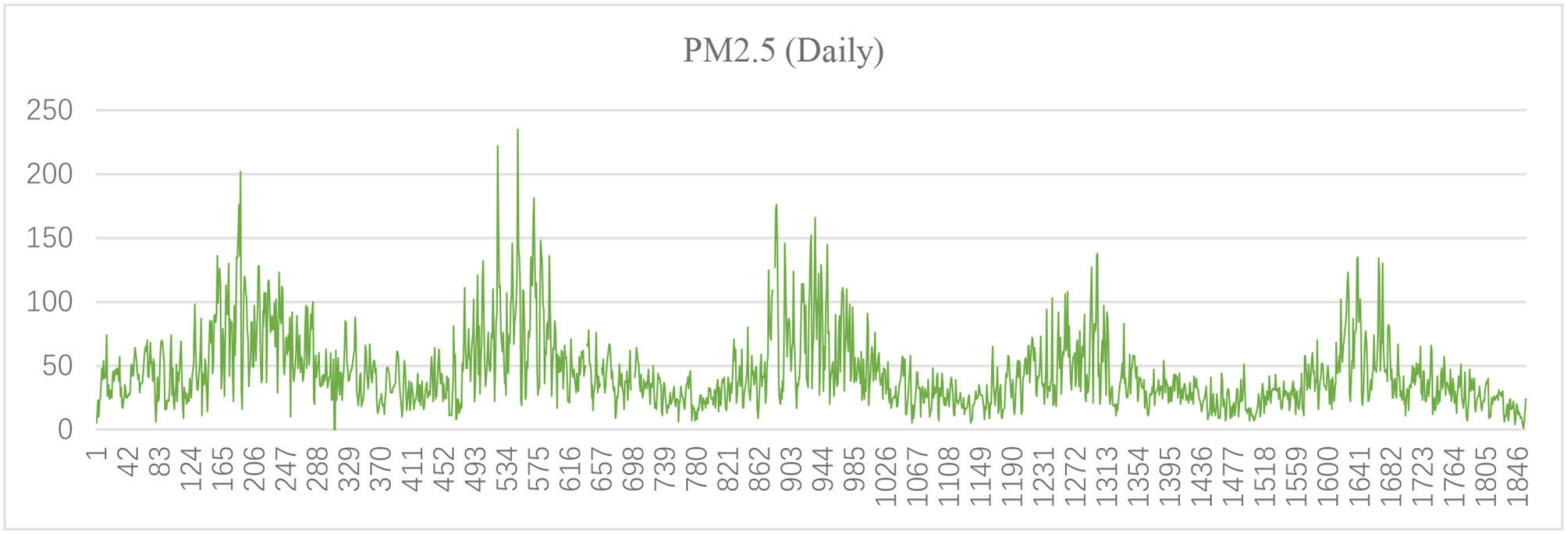
Daily PM2.5 of Hefei from July 2016 to July 2021 (μg/m^3^).

**Figure 2:**
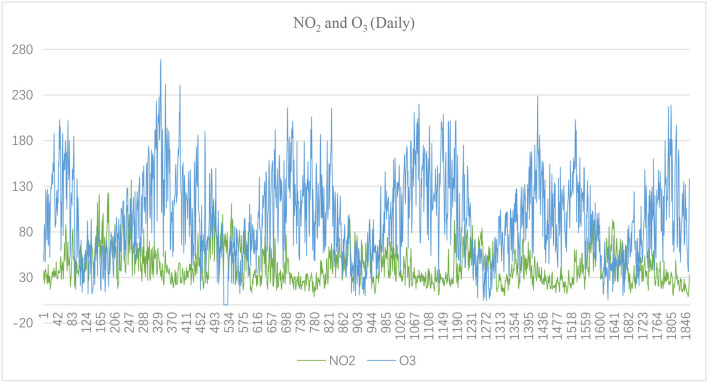
Daily NO_2_ and O_3_ of Hefei from July 2016 to July 2021(μg/m^3^).

**Figure 3:**
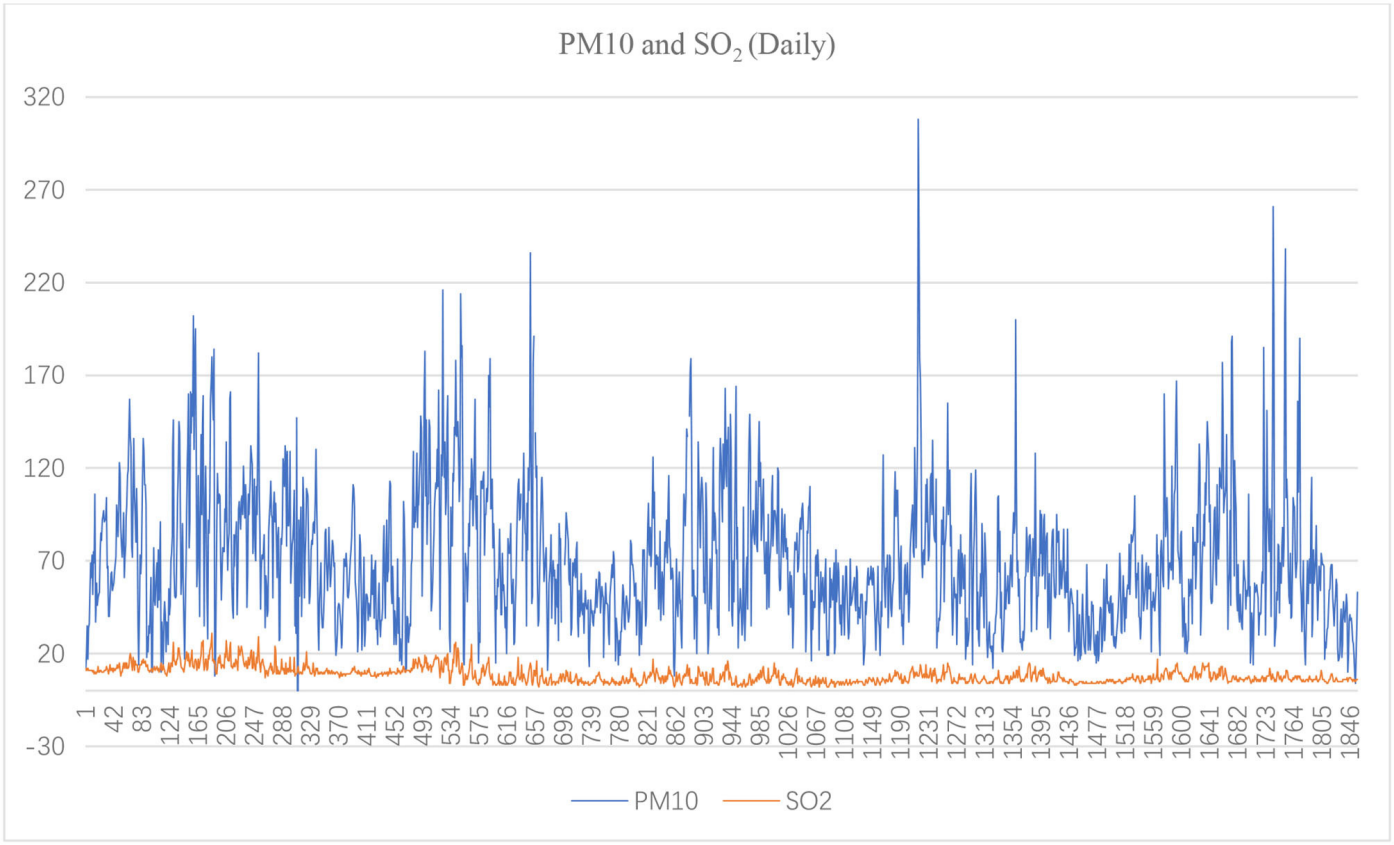
Daily PM10 and SO_2_ of Hefei from July 2016 to July 2021(μg/m^3^).

**Figure 4:**
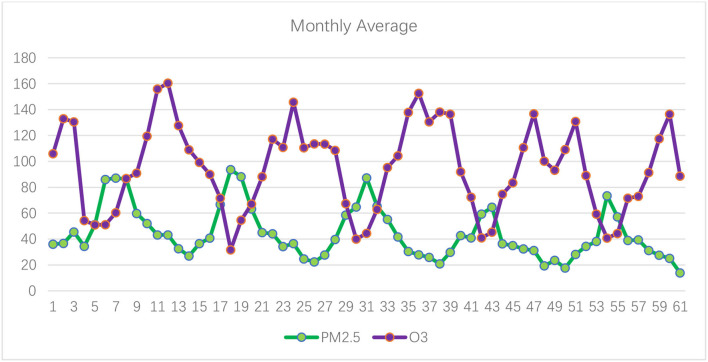
Monthly average of PM2.5 and O_3_ of Hefei from July 2016 to July 2021 (μg/m^3^).

**Figure 5:**
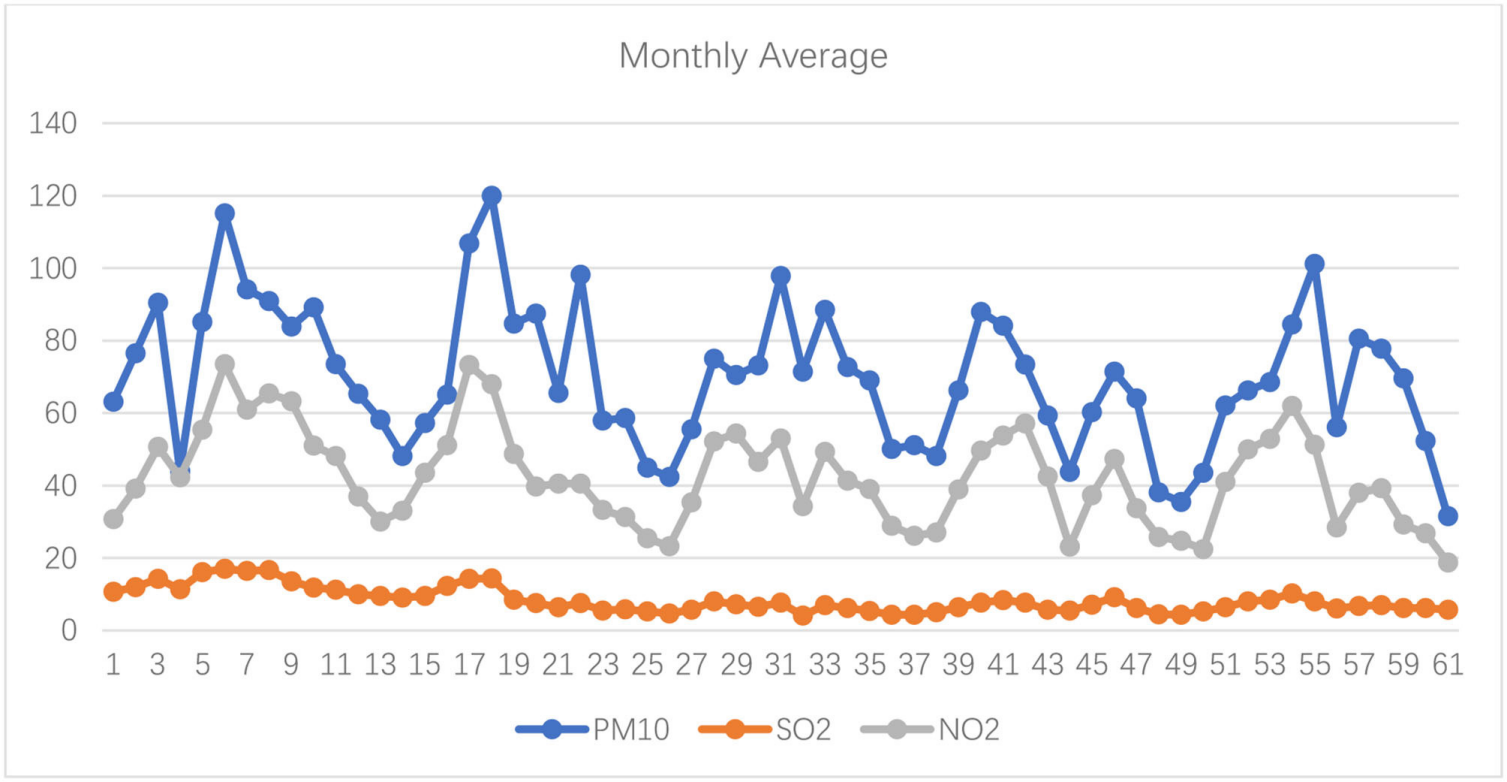
Monthly average of PM10, SO_2_, and NO_2_ of Hefei from July 2016 to July 2021 (μg/m^3^).

**Figure 6:**
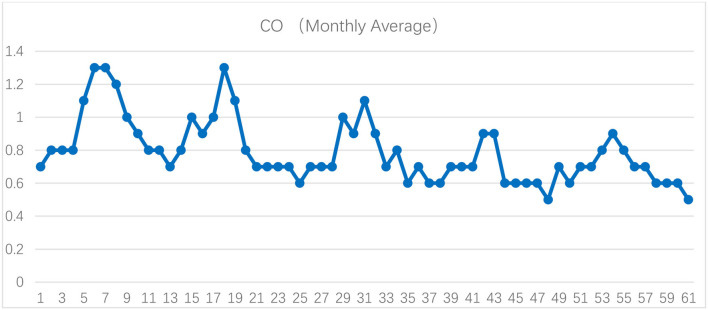
Monthly average of CO of Hefei from July 2016 to July 2021 (mg/m^3^).

Figures 1–3 show the daily data of each factor from July 2016 to July 2021, while Figures 4–6 present the monthly average data. From the Figures, we can find two observations: first, the data periodically fluctuate; and second, the periodicity of O_3_ is reversed with the other factors (see Figures 2, 4).

## Improved Double-weight Fuzzy Comprehensive Evaluation Method

A fuzzy comprehensive evaluation is a simple analysis, judgment, and evaluation of those problems with fuzziness by using the fuzzy mathematics principle and conversion between qualitative and quantitative problems by relying on membership degree theory. A fuzzy matrix is a model that reflects the influence of the membership degree of various factors on the evaluation grade. Membership degrees can be used as a numerical index and as a standard function of the evaluation index. In general, the membership degree of evaluation grade varies with the form of function. Generally speaking, it not only reflects a strong system, not to mention that the evaluation results are relatively clear, but it can also construct a model for fuzzy problems and provide solutions. In this section, we use the ID-FCE method. We particularly focus on the impact of the toxicity index, especially the impact of carbon monoxide.

According to the ambient Air Quality Standards (GB3095-2012), we choose PM_2.5_, PM_10_, SO_2_, CO, NO_2_, and O_3_ as the evaluation factors. For i=1, 2, 3, …, n, establishing evaluation factor set:


(1) $$\begin{array}{l} \text{u}=\left\{{\text{u}}_{1},{\text{u}}_{2},{\text{u}}_{3},\ldots ,{\text{u}}_{\text{n}}\right\} \end{array}$$

### Evaluation Grade

According to the ambient China Air Quality Standards (GB3095-2012) and China technical provisions of the ambient Air Quality Index (AQI), we classify air quality into j = 1, 2, 3, …, m grades. This article multiples slope member functions, the evaluation set ***V*** is then established,


(2) $$\begin{array}{l} V=\left\{\text{I},\text{II},\text{III},\text{IV},\text{V},\text{VI}\right\}, \end{array}$$

and the evaluation level is divided into six grades; see following Table 3.

**Table 3:** Multiple slope member function grade.

**Grade**	**I**	**II**	**III**	**IV**	**V**	**VI**
	**s**_**i1**_ **(1)** **−s**_**i1**_ **(2)**	**s**_**i2**_ **(1)** **−s**_**i2**_ **(2)**	**s**_**i3**_ **(1)** **−s**_**i3**_ **(2)**	**s**_**i4**_ **(1)** **−s**_**i4**_ **(2)**	**s**_**i5**_ **(1)** **−s**_**i5**_ **(2)**	**s**_**i6**_ **(1)** **−s**_**i6**_ **(2)**
PM_2.5_	0–35	35–75	75–115	115–150	150–250	250–∞
PM_10_	0–50	50–150	150–250	250–350	350–520	520–∞
SO_2_	0–50	50–150	150–475	475–800	800–1,600	1,600–∞
CO	0–2	2–4	4–14	14–24	24–36	36–∞
NO_2_	0–40	40–80	80–180	180–280	280–565	565–∞
O_3_	0–100	100–160	160–215	215–265	265–800	800-∞

### Member Function Matrix *R*

In this article, the slope of the right side is twice the slope of the left side. The following gives the evaluation a factor membership function for each evaluation grade.

The i-th evaluation factor to the j (j = 1) level membership function is


(3) $$\begin{array}{l} r_{ij}=\{\begin{array}{c} 1,\;S_{ij}\left(1\right)\;\leq x_{i}\leq S_{ij}\left(2\right)\\ 1-\frac{x_{i}-S_{ij}\left(2\right)}{S_{im}\left(1\right)},\;x_{i}>S_{ij}\left(2\right)\; \end{array} \end{array}$$

The i-th evaluation factor is relative to the th j (2≤j≤m-1) level membership function is


(4) $$\begin{array}{l} r_{ij}=\{\begin{array}{c} 1+\frac{x_{i}-S_{ij}\left(2\right)}{{2S}_{im}\left(1\right)},\;x_{i}\;<S_{ij}\left(1\right)\;\\ \;1,\;S_{ij}\left(1\right)\;\leq x_{i}\leq S_{ij}\left(2\right)\;\\ 1-\frac{x_{i}-S_{ij}\left(2\right)}{S_{im}\left(1\right)},\;x_{i}>S_{ij}\left(2\right)\; \end{array} \end{array}$$

The i-th evaluation factor for the j (j = m) level is


(5) $$\begin{array}{l} r_{ij}=\{\begin{array}{c} 1+\frac{x_{i}-S_{ij}\left(2\right)}{{2S}_{im}\left(1\right)}\;,\;x_{i}<S_{ij}\left(1\right)\;\\ \;\\ 1,\;S_{ij}\left(1\right)\;\leq x_{i}\leq S_{ij}\left(2\right)\; \end{array} \end{array}$$

S_ij_(1) and S_ij_(2), respectively, represent the lower threshold and the upper threshold of the j-th grade of the i-th evaluation factor, while S_im_(1) represents the lower threshold of the m-th grade (the highest level) of the i-th pollution factor. When r_ij_ appears negative, it is stipulated that r_ij_ = 0.

The member function of PM2.5 (where *x*_1_ is the observation value) is:


(6) $$\begin{array}{lll} r_{11} & = & \{\begin{array}{c} 1,\;0\;\leq x_{1}\leq 35\\ 1-\frac{x_{1}-35}{250},\;x_{1}>35\; \end{array} \end{array}$$

(7) $$\begin{array}{lll} r_{12} & = & \{\begin{array}{c} 1+\frac{x_{1}-35}{2\times 250},\;x_{1}\;<35\;\\ \;1\;35\;\leq x_{1}\leq 75\;\\ 1-\frac{x_{1}-75}{250},\;x_{1}>75\; \end{array} \end{array}$$

(8) $$\begin{array}{lll} r_{13} & = & \{\begin{array}{c} 1+\frac{x_{1}-75}{2\times 250},\;x_{1}\;<75\;\\ \;1,\;75\;\leq x_{1}\leq 115\;\\ 1-\frac{x_{1}-115}{250},\;x_{1}>115\; \end{array} \end{array}$$

(9) $$\begin{array}{lll} r_{14} & = & \{\begin{array}{c} 1+\frac{x_{1}-115}{2\times 250},\;x_{1}\;<115\;\\ \;1,\;115\;\leq x_{1}\leq 150\;\\ 1-\frac{x_{1}-150}{250},\;x_{1}>150\; \end{array} \end{array}$$

(10) $$\begin{array}{lll} r_{15} & = & \{\begin{array}{c} 1+\frac{x_{1}-150}{2\times 250},\;x_{1}\;<150\;\\ \;1,\;150\;\leq x_{1}\leq 250\;\\ 1-\frac{x_{1}-250}{250},\;x_{1}>250\; \end{array} \end{array}$$

(11) $$\begin{array}{lll} r_{16} & = & \{\begin{array}{c} 1+\frac{x_{1}-250}{2\times 250},\;x_{1}<250\;\\ 1,\;x_{1}\geq 250\; \end{array} \end{array}$$

Applying the same method, other evaluation factors are calculated according to the evaluation grade of the multiple slope membership function and the above membership function. The matrix of the membership function is as follows:


(12) $$\begin{array}{l} R=\left[\begin{array}{cccccc} r_{11} & r_{12} & r_{13} & r_{14} & r_{15} & r_{16}\\ r_{21} & r_{22} & r_{23} & r_{24} & r_{25} & r_{26}\\ r_{31} & r_{32} & r_{33} & r_{34} & r_{35} & r_{36}\\ r_{41} & r_{42} & r_{43} & r_{44} & r_{45} & r_{46}\\ r_{51} & r_{52} & r_{53} & r_{54} & r_{55} & r_{56}\\ r_{61} & r_{62} & r_{63} & r_{64} & r_{65} & r_{66} \end{array}\right] \end{array}$$

### Factor Weight and Evaluation Solution

We first give the weight of the six factors, namely, PM_2.5_, PM_10_, SO_2_, CO, NO_2_, and O_3_. Then, the overweight method is adopted to determine that the weight and the toxicity index of the evaluation factors are added, which is shown in Table 1. After exceeding the standard method, toxicity calculation is carried out, and the formula is as follows:


(13) $$\begin{array}{l} c_{i}=\left(x_{i}/\sum_{i=1}^{5}S_{ij}/5\right)/\sum_{i=1}^{6}\left[x_{i}/\left(\sum_{i=1}^{5}S_{ij}/5\right)\right] \end{array}$$

(14) $$\begin{array}{l} a_{i}=\frac{c_{i}}{f_{i}}/\sum_{i=1}^{6}\frac{c_{i}}{f_{i}} \end{array}$$

The weight set is


(15) $$\begin{array}{l} A=\left(a_{1},a_{2},a_{3},a_{4},a_{5},a_{6}\;\right) \end{array}$$

After the weight set is given, we can calculate the fuzzy evaluation result:


(16) $$\begin{array}{l} B=A.R=\left(a_{1},a_{2},a_{3},a_{4},a_{5},a_{6}\right)\;[\begin{array}{cccccc} r_{11} & r_{12} & r_{13} & r_{14} & r_{15} & r_{16}\\ r_{21} & r_{22} & r_{23} & r_{24} & r_{25} & r_{26}\\ r_{31} & r_{32} & r_{33} & r_{34} & r_{35} & r_{36}\\ r_{41} & r_{42} & r_{43} & r_{44} & r_{45} & r_{46}\\ r_{51} & r_{52} & r_{53} & r_{54} & r_{55} & r_{56}\\ r_{61} & r_{62} & r_{63} & r_{64} & r_{65} & r_{66} \end{array}]\\ \;=\left(b_{1},b_{2},b_{3},b_{4},b_{5},b_{6}\right) \end{array}$$

According to the principle of maximum membership degree, we make the comparison of *b*_1_*, b*_2_*, b*_3_*, b*_4_*, b*_5_, and *b*_6_, then find the maximum. If *b*_*j*_ = max*{b*_1_*, b*_2_*, b*_3_*, b*_4_*, b*_5_*, b*_6_*},j* = *1,2,…,6*, then we say the evaluation grade is *j*.

## Case Study

This article uses the daily air quality data of Hefei City from 2016 to 2021 for fuzzy evaluation analysis. The air quality index data came from the China Air Quality Online Monitoring and Analysis Platform (https://www.aqistudy.cn). The unit of CO is mg/m^3^, and the unit for the other five pollutants is μg/m^3^. We take the data of July 2016 as an example to analyze its air quality level.

According to Table 4, the following calculation is based on the July 1, 2016 data.

(a) By the formulas (3), (4), and (5), we can find the following membership matrix

(17) $$\begin{array}{l} R=\left[\begin{array}{cccccc} 1 & .94 & .86 & .78 & .71 & .51\\ 1 & .95 & .84 & .72 & .60 & .51\\ 1 & .99 & .96 & .86 & .75 & .50\\ 1 & .98 & .95 & .81 & .68 & .51\\ 1 & .99 & .95 & .86 & .78 & .52\\ 1 & .97 & .94 & .90 & .87 & .54 \end{array}\right]. \end{array}$$

(b) By Table 1 and formula (13), we can find the weight set C.

**Table 4:** Daily air quality pollutant concentration in Hefei City from July 2016 to July 2021.

**Date**	**PM_**2.5**_**	**PM_**10**_**	**SO_**2**_**	**CO**	**NO_**2**_**	**O_**3**_**
2016/7/1	5	12	11	0.6	27	56
2016/7/2	12	20	12	0.7	37	48
2016/7/3	23	35	11	0.8	31	88
2016/7/4	10	17	12	0.6	23	79
2016/7/5	23	34	11	0.7	35	79
2016/7/6	24	36	11	0.8	39	47
2016/7/26	47	94	10	0.9	36	132
2016/7/27	43	97	10	0.7	36	133
2016/7/28	48	91	11	0.8	30	155
2016/7/29	45	92	11	0.7	32	147
2016/7/30	38	93	11	0.7	40	146
2016/7/31	57	104	13	0.8	36	188
2021/7/1	30	57	6	0.7	22	156
2021/7/2	14	23	5	0.6	19	58
2021/7/3	6	16	5	0.5	15	96
2021/7/4	11	17	5	0.6	18	91
2021/7/5	10	20	5	0.8	29	47
2021/7/6	18	36	5	0.7	25	62
2021/7/26	7	18	6	0.4	12	46
2021/7/27	3	8	5	0.4	9	38
2021/7/28	1	4	5	0.4	10	36
2021/7/29	8	22	6	0.4	17	69
2021/7/30	17	36	6	0.6	23	112
2021/7/31	24	53	6	0.7	33	138

Since PM_2.5_ = 5 on the day of July 1, 2016 then


(18) $$\begin{array}{l} c_{1}=\;\left(5/125\right)/0.8928=0.0448. \end{array}$$

Similarly, the weight of PM_10_,SO_2_,CO, NO_2_, and O_3_, respectively, are


$$\begin{array}{lll} c_{2} & = & \left(12/264\right)/0.4227=0.1075,\\ c_{3} & = & \left(11/615\right)/0.1815=0.0985,\\ c_{4} & = & \left(0.6/80\right)/1.395=0.0054,\\ c_{5} & = & \left(27/229\right)/0.4873=0.2420,\\ c_{6} & = & \left(56/308\right)/0.3623=0.5018. \end{array}$$

According to Table 2 and formula (14), the weight set A is obtained,


$$\begin{array}{lll} a_{1} & = & \frac{.0448}{1}/0.3471=0.1291,\\ a_{2} & = & \frac{.1075}{2}/0.3471=0.1549,\\ a_{3} & = & \frac{.0985}{5}/0.3471=0.0568,\\ a_{4} & = & \frac{.0054}{5}/0.3471=0.0031,\\ a_{5} & = & \frac{.2420}{4}/0.3471=0.1743,\\ a_{6} & = & \frac{.5018}{3}/0.3471=0.4819. \end{array}$$

Therefore, we have *A* = (.1291, 0.1549, 0.0568, 0.0031, 0.1743, and 0.4819).

(c) By formula (16) we have the evaluation result

$$\begin{array}{l} B=A\times R\\ \;=\left(.1291,\;0.1549,\;0.0568,\;0.0031,\;0.1743,\;0.4819\right)\\ \;\left[\begin{array}{cccccc} 1 & .94 & .86 & .78 & .71 & .51\\ 1 & .95 & .84 & .72 & .60 & .51\\ 1 & .99 & .96 & .86 & .75 & .50\\ 1 & .98 & .95 & .81 & .68 & .51\\ 1 & .99 & .95 & .86 & .78 & .52\\ 1 & .97 & .94 & .90 & .87 & .54 \end{array}\right]\\ \;=\left(1,\;0.97,\;0.92,\;0.85,\;0.78,\;and\;0.53\right). \end{array}$$

Since *b*_1_=max*{b*_1_*, b*_2_*, b*_3_*, b*_4_*, b*_5_*, b*_6_*}*, then according to the above result, we know that the air quality on the day of July 1, 2016 is the first grade based on the improved double-weight fuzzy comprehensive evaluation method. Similarly, we can get the evaluation results on July 31, 2020, in which the membership matrix *R* and the weight set *A* and *B* are, respectively,


$$\begin{array}{lll} R & = & \left[\begin{array}{cccccc} 1 & .98 & .90 & .82 & .75 & .55\\ .99 & 1 & .89 & .77 & .65 & .56\\ 1 & .99 & .96 & .85 & .75 & .50\\ 1 & .98 & .95 & .82 & .68 & .51\\ 1 & .99 & .96 & .87 & .78 & .53\\ .95 & 1 & .99 & .95 & .92 & .59 \end{array}\right],\\ \text{A} & = & \left(0.3866,0.2187,0.0039,0.0176,0.0725,\text{and }0.3007\right),\\ \text{B} & = & \left(0.98,0.99,0.92,0.85,0.78,\text{and }0.56\right), \end{array}$$

which implies that *b*_2_ is the largest value of *{b*_1_*, b*_2_*, b*_3_*, b*_4_*, b*_5_*, b*_6_*}*, then the air quality on July 31, 2021 is the second grade based on the improved double-weight fuzzy comprehensive evaluation method.

After applying the same calculation, we have the following ID-FCE results (see Tables 5, 6 for December and July of 2016–2021).

**Table 5:** Daily grade results based on Improved Double-weighted Fuzzy Comprehensive Evaluation (ID-FCE; December and July of 2016–2021).

**Date (2016)**	**Grade**	**Date (2016)**	**Grade**	**Date (2017)**	**Grade**	**Date (2017)**	**Grade**	**Date (2018)**	**Grade**	**Date (2018)**	**Grade**
July/1	I	Dec/1	III	July/1	I	Dec/1	II	July/1	II	Dec/1	V
July/2	I	Dec/2	III	July/2	I	Dec/2	III	July/2	II	Dec/2	IV
July/3	I	Dec/3	III	July/3	I	Dec/3	III	July/3	II	Dec/3	III
July/4	I	Dec/4	III	July/4	I	Dec/4	V	July/4	II	Dec/4	I
July/5	I	Dec/5	IV	July/5	I	Dec/5	V	July/5	I	Dec/5	I
July/6	I	Dec/6	III	July/6	I	Dec/6	III	July/6	I	Dec/6	I
July/7	II	Dec/7	III	July/7	I	Dec/7	III	July/7	I	Dec/7	I
July/8	II	Dec/8	IV	July/8	I	Dec/8	II	July/8	II	Dec/8	I
July/9	II	Dec/9	III	July/9	I	Dec/9	II	July/9	I	Dec/9	II
July/10	II	Dec/10	I	July/10	I	Dec/10	II	July/10	I	Dec/10	I
July/11	II	Dec/11	II	July/11	I	Dec/11	III	July/11	I	Dec/11	II
July/12	II	Dec/12	II	July/12	I	Dec/12	II	July/12	I	Dec/12	IV
July/13	II	Dec/13	II	July/13	II	Dec/13	II	July/13	I	Dec/13	III
July/14	II	Dec/14	I	July/14	II	Dec/14	II	July/14	I	Dec/14	II
July/15	I	Dec/15	II	July/15	II	Dec/15	I	July/15	I	Dec/15	II
July/16	I	Dec/16	III	July/16	II	Dec/16	III	July/16	II	Dec/16	III
July/17	II	Dec/17	III	July/17	II	Dec/17	II	July/17	I	Dec/17	III
July/18	I	Dec/18	III	July/18	I	Dec/18	II	July/18	I	Dec/18	III
July/19	I	Dec/19	III	July/19	I	Dec/19	II	July/19	II	Dec/19	II
July/20	I	Dec/20	IV	July/20	II	Dec/20	II	July/20	II	Dec/20	II
July/21	I	Dec/21	II	July/21	II	Dec/21	III	July/21	II	Dec/21	II
July/22	II	Dec/22	III	July/22	II	Dec/22	III	July/22	I	Dec/22	II
July/23	II	Dec/23	II	July/23	II	Dec/23	IV	July/23	I	Dec/23	III
July/24	II	Dec/24	III	July/24	II	Dec/24	III	July/24	II	Dec/24	II
July/25	II	Dec/25	II	July/25	II	Dec/25	II	July/25	I	Dec/25	II
July/26	II	Dec/26	I	July/26	II	Dec/26	III	July/26	I	Dec/26	I
July/27	II	Dec/27	III	July/27	II	Dec/27	III	July/27	I	Dec/27	I
July/28	II	Dec/28	II	July/28	II	Dec/28	III	July/28	I	Dec/28	I
July/29	II	Dec/29	II	July/29	II	Dec/29	III	July/29	II	Dec/29	II
July/30	II	Dec/30	IV	July/30	II	Dec/30	V	July/30	II	Dec/30	II
July/31	II	Dec/31	IV	July/31	I	Dec/31	IV	July/31	II	Dec/31	II

**Table 6:** Daily grade results based on ID-FCE (December and July of 2016–2021, continued).

**Date (2019)**	**Grade**	**Date (2019)**	**Grade**	**Date (2020)**	**Grade**	**Date (2020)**	**Grade**	**Date (2021)**	**Grade**
July/1	II	Dec/1	II	July/1	II	Dec/1	II	July/1	II
July/2	II	Dec/2	III	July/2	II	Dec/2	II	July/2	I
July/3	II	Dec/3	II	July/3	I	Dec/3	III	July/3	I
July/4	II	Dec/4	II	July/4	I	Dec/4	II	July/4	I
July/5	II	Dec/5	II	July/5	I	Dec/5	II	July/5	I
July/6	I	Dec/6	I	July/6	I	Dec/6	II	July/6	I
July/7	II	Dec/7	II	July/7	I	Dec/7	II	July/7	I
July/8	I	Dec/8	II	July/8	II	Dec/8	II	July/8	I
July/9	I	Dec/9	II	July/9	I	Dec/9	III	July/9	I
July/10	II	Dec/10	II	July/10	II	Dec/10	III	July/10	I
July/11	II	Dec/11	III	July/11	I	Dec/11	III	July/11	I
July/12	I	Dec/12	II	July/12	I	Dec/12	III	July/12	I
July/13	I	Dec/13	II	July/13	I	Dec/13	III	July/13	I
July/14	II	Dec/14	III	July/14	I	Dec/14	I	July/14	I
July/15	II	Dec/15	II	July/15	I	Dec/15	I	July/15	II
July/16	II	Dec/16	II	July/16	I	Dec/16	II	July/16	I
July/17	II	Dec/17	II	July/17	I	Dec/17	II	July/17	I
July/18	I	Dec/18	I	July/18	I	Dec/18	II	July/18	I
July/19	I	Dec/19	I	July/19	I	Dec/19	III	July/19	I
July/20	I	Dec/20	II	July/20	II	Dec/20	II	July/20	I
July/21	II	Dec/21	II	July/21	I	Dec/21	II	July/21	I
July/22	II	Dec/22	II	July/22	I	Dec/22	II	July/22	I
July/23	I	Dec/23	II	July/23	I	Dec/23	III	July/23	I
July/24	I	Dec/24	I	July/24	I	Dec/24	IV	July/24	I
July/25	I	Dec/25	II	July/25	II	Dec/25	IV	July/25	I
July/26	I	Dec/26	II	July/26	II	Dec/26	III	July/26	I
July/27	II	Dec/27	II	July/27	I	Dec/27	III	July/27	I
July/28	II	Dec/28	II	July/28	II	Dec/28	III	July/28	I
July/29	II	Dec/29	I	July/29	II	Dec/29	II	July/29	II
July/30	II	Dec/30	II	July/30	II	Dec/30	I	July/30	II
July/31	II	Dec/31	I	July/31	I	Dec/31	I	July/31	II

Now, we turn to test the monthly grade results based on the improved double-weight fuzzy comprehensive evaluation method (Table 7). The monthly average value of PM_2.5_, PM_10_, SO_2_, CO, NO_2_, and O_3_ are shown in Figures 4–6. From Table 7, we can find that, from the monthly perspective, the overall air quality conditions are good, most of the grades are I or II, and only 1 month (December 2017) is grade III. Furthermore, the air quality in December and January is always not good.

**Table 7:** Monthly grade results based on ID-FCE (July 2016–July 2021).

**Month (2016)**	**Grade**	**Month (2017)**	**Grade**	**Month (2018)**	**Grade**	**Month (2019)**	**Grade**	**Month (2020)**	**Grade**	**Month (2021)**	**Grade**
		1	II	1	II	1	II	1	II	1	II
		2	II	2	II	2	II	2	I	2	II
		3	II	3	II	3	II	3	II	3	II
		4	II	4	II	4	II	4	II	4	II
		5	II	5	II	5	II	5	II	5	II
		6	II	6	II	6	II	6	I	6	II
7	II	7	II	7	I	7	II	7	I	7	I
8	II	8	I	8	I	8	II	8	I		
9	II	9	II	9	II	9	II	9	II		
10	I	10	II	10	II	10	II	10	II		
11	II	11	II	11	II	11	II	11	II		
12	II	12	**III**	12	II	12	II	12	II		

**Figure 7:**
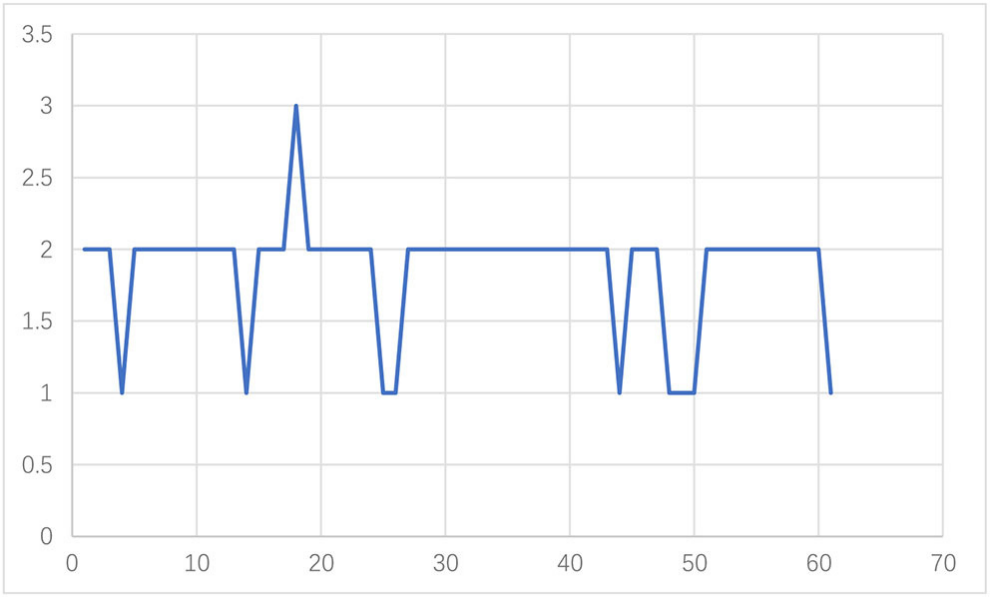
Monthly grade results based on ID-FCE (July 2016-July 2021).

Figure 7 shows the monthly grade results based on the ID-FCE of July 2016-July 2021. From Figure 7, Table 6, we can find that, with the emphasis of the government on air quality in recent years, the air quality has become better (the grade becomes lower) from 2016 to 2021.

## Conclusion

Hefei is an innovative city in Anhui Province. In recent years, to reduce carbon emissions and air pollution, Hefei has vigorously introduced new energy vehicles. Many auto manufacturers, including NIO, Volkswagen, and BYD, have set up factories in Hefei. In this article, we analyzed the air quality of Hefei City of Anhui Province from July 2016 to 2021 using an ID-FCE method. According to the model, we evaluate the grades of both the daily air quality and the monthly air quality. We focus on the impact of the toxicity index, especially the impact of carbon monoxide, which is an essential index in automobile exhaust emission detection. The ID-FCE can evaluate well the air quality based on our three observations.

First, the analysis shows that from July 2016 to July 2021, with the increasing awareness of environmental protection and the attention of the government to the quality of air in recent years, the air quality gradually became better (the grades become lower).

Second, the value of the factors PM_2.5_, PM_10_, SO_2_, CO, NO_2_, and O_3_ fluctuate periodically from year to year. In addition, the periodicity of O_3_ is reversed with the other factors.

Finally, from the monthly average perspective, the overall air quality is good. Most of the grades are I-II, and only 1 month (December 2017) is grade III. Furthermore, the air quality in the winter (especially in December and January) is always not good.

In line with Huang et al. (21), our article also shows that temperature has positively or negatively impacted air pollution (21). Specifically, in the spring, summer, and autumn seasons, the air quality is always good but is reversed in winter. However, different from Lu et al. (7, 13, 16), Sun et al. (22, 23), and Yu et al. (24), our article indicates that air quality has gradually improved in recent years due to environmental protection of people and the air quality improvement by the government.

## Data Availability Statement

The original contributions presented in the study are included in the article/supplementary material, further inquiries can be directed to the corresponding author.

## Author Contributions

WP and XL: formal analysis. FT-H: investigation and validation. WP: methodology and writing original draft. XL: software. WP and FT-H: writing - review ad editing, All authors have read and agreed to the published version of the manuscript.

## Funding

This research was supported by Anhui Province Social Science Innovation Development Research Project (2021CX077), University Key Project of Natural Science Foundation of Anhui Province (No. KJ2021A1031, KJ2019A0683), the University Outstanding Young Talents Project of Anhui Province (No. gxyq2021018, gxyq2019082), Key Project of Natural Science Foundation of Chaohu Univesity (No. XLZ-201801) and the Grant in aid for Excellent Young Researcher of the Ministry of Education of Japan.

## Conflict of Interest

The authors declare that the research was conducted in the absence of any commercial or financial relationships that could be construed as a potential conflict of interest.

## Publisher's Note

All claims expressed in this article are solely those of the authors and do not necessarily represent those of their affiliated organizations, or those of the publisher, the editors and the reviewers. Any product that may be evaluated in this article, or claim that may be made by its manufacturer, is not guaranteed or endorsed by the publisher.
